# Omicron Spike confers enhanced infectivity and interferon resistance to SARS-CoV-2 in human nasal tissue

**DOI:** 10.1101/2023.05.06.539698

**Published:** 2023-05-08

**Authors:** Guoli Shi, Tiansheng Li, Kin Kui Lai, Jonathan W Yewdell, Alex A Compton

**Affiliations:** 1 HIV Dynamics and Replication Program, Center for Cancer Research, National Cancer Institute, Frederick, MD; 2 Laboratory of Viral Diseases, National Institute of Allergy and Infectious Diseases, Bethesda, MD

## Abstract

Omicron emerged following COVID-19 vaccination campaigns, displaced previous SARS-CoV-2 variants of concern worldwide, and gave rise to lineages that continue to spread. Here, we show that Omicron exhibits increased infectivity in primary adult upper airway tissue. Using recombinant forms of SARS-CoV-2 and nasal epithelial cells cultured at the liquid-air interface, enhanced infectivity maps to the step of cellular entry and evolved recently through mutations unique to Omicron Spike. Unlike earlier variants of SARS-CoV-2, Omicron enters nasal cells independently of serine transmembrane proteases and instead relies upon matrix metalloproteinases to catalyze membrane fusion. This entry pathway unlocked by Omicron Spike enables evasion of interferon-induced factors that restrict SARS-CoV-2 entry following attachment. Therefore, the increased transmissibility exhibited by Omicron in humans may be attributed not only to its evasion of vaccine-elicited adaptive immunity, but also to its superior invasion of nasal epithelia and resistance to the cell-intrinsic barriers present therein.

## Introduction

Efficient infection of human cells by severe acute respiratory syndrome coronavirus 2 (SARS-CoV-2) requires the interaction of SARS-CoV-2 Spike with its receptor at the cell surface, angiotensin-converting enzyme 2 (ACE2)^1,2^. In addition, Spike has been described to bind to various cellular factors to promote coronavirus attachment to the cell surface. 78-kilodalton glucose-regulated protein, neurolipin-1, high-density lipoprotein scavenger receptor B type 1, CD209/CD299, Axl, sialic acid, and heparan sulfate have all been reported to interact with Spike and promote the virus entry process^3–9^. In general, it is thought that factors aiding virus attachment enable subsequent ACE2 binding. Engagement of ACE2 alters Spike conformation and facilitates its processing by cellular proteases, such as serine transmembrane proteases like TMPRSS2, matrix metalloproteinases (MMPs), or a disintegrin and metalloproteinases (ADAMs)^2,10–14^. Protease cleavage enables Spike to trigger fusion between viral and cellular membranes to complete cellular entry^15^.

The Omicron B.1.1.529.1 (BA.1) variant emerged in late 2021 and, as a result of increased transmissibility between humans^16,17^, quickly replaced the Delta variant of concern as the dominant form of SARS-CoV-2 circulating worldwide (https://www.cdc.gov/coronavirus/2019-ncov/variants/variant-classifications.html). Compared to previous SARS-CoV-2 variants of concern, Spike from BA.1 contained a plethora of unique mutations, and its descendants (including the BA.2, BA.4, BA.5, BQ.1, and XBB subvariants) contained additional mutations in Spike (https://covid.cdc.gov/covid-data-tracker/#datatracker-home). The functional characterization of Omicron has revealed that said Spike mutations, including those found in the receptor binding domain, may allow for improved affinity for ACE2 and/or evasion of neutralizing antibodies induced by COVID-19 vaccination^18–26^. However, it remains unclear whether antibody evasion and ACE2 affinity are the only factors explaining the improved transmissibility of Omicron subvariants. In fact, Omicron Spike exhibits similar affinity for ACE2 compared to the early D614G variant^23,27^, suggesting that Omicron Spike enables improved virus transmissibility for other reasons.

It has also been reported that Omicron utilizes a distinct cellular entry pathway compared to ancestral or early forms of SARS-CoV-2^28–32^. Specifically, the proteolytic activation of Omicron Spike is less dependent on TMPRSS2 but may be more dependent on cathepsin activity in endolysosomes or MMP activity at the plasma membrane^11^. However, the precise characterization of the entry pathways used by Omicron in physiologically relevant primary cells is lacking, and whether they are sensitive to cell-intrinsic antiviral barriers has not been determined. The apparent loss of TMPRSS2 dependence may have consequences for the cellular tropism of Omicron in vivo. Some reports suggest that Omicron BA.1 exhibits a decreased propensity for lower respiratory tract (lung) infection, which may explain the decreased pathogenicity associated with Omicron infections^33–38^. Since the upper respiratory tract, and specifically the nasal epithelium, represents the initial site of SARS-CoV-2 infection and the initial protective innate immune response^39,40^, we compared the ability of Omicron to invade human nasal epithelia relative to ancestral SARS-CoV-2 and the preceding variant of concern, Delta, using a primary human nasal tissue culture model. We found that Omicron BA.1 and BA.2 exhibited a markedly enhanced infectivity in primary nasal epithelia compared to early SARS-CoV-2, and using recombinant virus, we show that Spike from Omicron, but not Delta, controled this phenotype. Furthermore, despite Omicron eliciting a type-I interferon response in nasal tissue, Omicron Spike used an MMP-mediated entry pathway that was associated with evasion of interferon-inducible factors targeting virus entry. Collectively, these findings may explain the increased transmissibility of Omicron as well as its ability to displace more interferon-sensitive variants of SARS-CoV-2.

Overall, our findings suggest that the global spread of Omicron was fueled not only by increased ACE2 affinity and resistance to vaccine-induced adaptive immunity, but also by resistance to nasal cell-intrinsic entry inhibitors.

## Results

**Figure 1.**
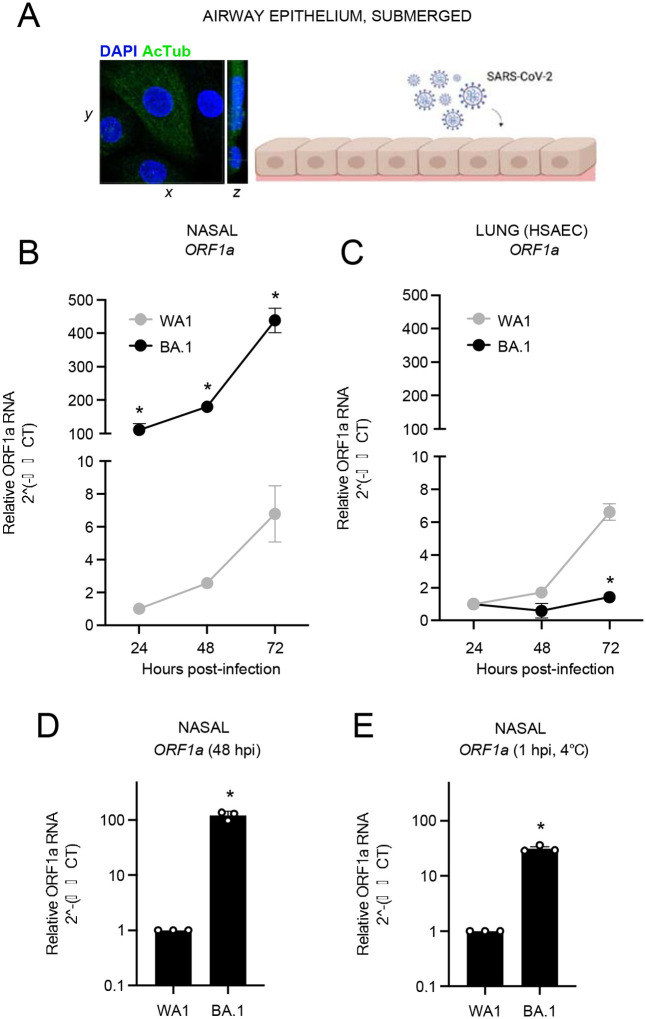
Omicron exhibits superior replicative fitness relative to early/ancestral SARS-CoV-2 in primary human nasal epithelial cells. (A) Primary human nasal epithelial cells (pooled from 3 human donors) were cultured as undifferentiated, submerged monolayers and challenged with SARS-CoV-2. Acetylated tubulin levels were determined by anti-AcTub immunofluorescence and DAPI was used to stain nuclei. 3D reconstructions of *xy* and *yz* fields are shown. (B) Primary human nasal epithelial cells (cells from three human donors, pooled) or (C) human small air way (lung) epithelial cells (single donor) were challenged with replication competent SARS-CoV-2 WA1 or Omicron BA.1 at an MOI of 0.05. Total cellular RNA was extracted and viral ORF1a was quantified by RT-qPCR at the indicated time points. Relative viral RNA abundance compared to actin was determined by the 2^(-ΔΔCT) method. ORF1a abundance of WA1 at 24 hours post inoculation was set to 1. Results are represented as means plus standard error from three independent infections. (D) Virus replication was measured by detecting ORF1a levels with RT-qPCR at 48 hours post inoculation with WA1 or BA.1 at an MOI of 0.05. (E) Virus attachment to cells was measured by detecting ORF1a levels with RT-qPCR at 1 hour post inoculation with WA1 or BA.1 at an MOI of 0.05 on ice. Results are presented as means plus standard error from two independent RT-qPCR runs. Statistically significant differences (* *P* < 0.05) between BA.1 and the corresponding data point of WA.1 were determined by one-way ANOVA. Cartoon made with Biorender.com.

Primary human nasal epithelial cells isolated from three donors were pooled and cultured as submerged monolayers, which allows for the propagation of cells in the basal (undifferentiated) state. Immunofluorescence staining for acetylated tubulin confirmed the absence of mature cilia in cells cultured in this manner (Figure 1A). Inoculation of nasal monolayers with early/ancestral SARS-CoV-2 (USA-WA1/2020 strain, herein referred to as WA1) or Omicron BA.1 and quantitation of virus replication by RT-qPCR of viral RNA at multiple time points revealed that BA.1 exhibited a superior replicative ability in these cells (Figure 1B). In contrast, BA.1 did not replicate in submerged monolayers of human small airway (lung) epithelial cells, while WA1 replicated with similar kinetics in nasal and lung cells (Figure 1C). These results suggest that BA.1 may exhibit a growth advantage that is specific to the upper airway of the human respiratory tract. Mechanistically, we found that the more than 100-fold replication advantage of BA.1 in nasal cells (Figure 1D) was accompanied by an approximately 30-fold superior capacity to adhere to the nasal cell surface (Figure 1E). Therefore, the enhanced replicative potential of BA.1 in nasal cells is associated with improved cell surface adhesion by Omicron Spike. During the preparation of this manuscript, it was reported that Omicron possesses greater adhesion to human nasal cells, and this was attributed to enhanced binding to cilia in differentiated nasal epithelia^41^. Our results here suggest that, besides cilia, other factors intrinsic to nasal cells must govern the improved replicative fitness of BA.1 compared to ancestral SARS-CoV-2.

**Figure 2.**
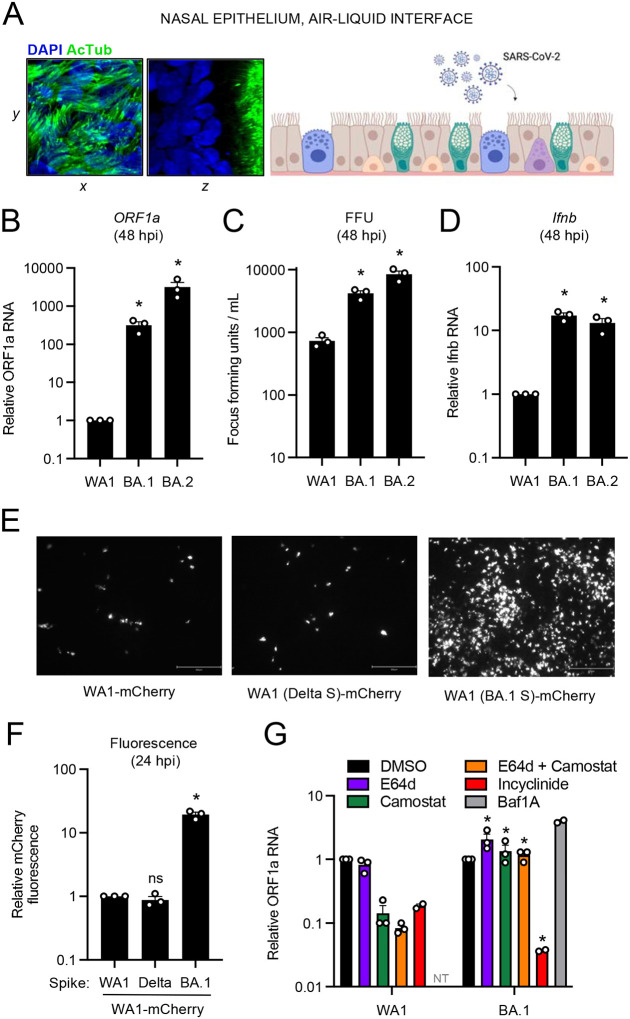
Omicron Spike enables enhanced infectivity in primary nasal epithelia cultured at the air-liquid interface and utilizes an MMP-dependent entry route. (A) Primary human nasal epithelial cells (pooled from 14 human donors) were cultured at the air-liquid interface to mimic the human respiratory tract and challenged with SARS-CoV-2. Acetylated tubulin levels were determined by anti-AcTub immunofluorescence and DAPI was used to stain nuclei. 3D reconstructions of *xy* and *yz* fields are shown. (B) Cells were subjected to total cellular RNA extraction at 48 hours post inoculation with 10000 plaque forming units of WA1, BA.1, or BA.2, and RT-qPCR of viral ORF1a was performed. Relative ORF1a transcript abundance compared to actin was determined by the 2^(-ΔΔCT) method. Symbols represent independent RT-qPCR runs. (C) Infectious virus titers from cell culture medium were measured by challenging Vero cells followed by fixation and immunostaining with anti-N antibody. Infectious units were quantified by measuring focus forming units with high-content imaging. Symbols represent results from three independent infections of Vero cells. (D) RT-qPCR of cellular Ifnb was performed at 48 hours post inoculation. Relative Ifnb transcript abundance compared to actin was determined by the 2^(-ΔΔCT) method. Symbols represent independent RT-qPCR runs. (E) Recombinant WA.1 encoding mCherry and Spike protein from WA1, Delta, or BA.1 were used to inoculate nasal ALI and infection was measured at 24 hours by mCherry fluorescence. Representative fields of view are shown. Scale bars = 300 μm. (F) Summary mCherry fluorescence from three independent infections is shown. The fluorescence intensity of WA1 (WA1 Spike)-mCherry was set to 1. (G) Nasal ALI were pretreated with 10 μM E64d, 10 μM camostat mesylate, a combination of 10 μM E64d and 10 μM camostat mesylate, 10 μM incyclinide, or 1 μM bafilomycin A1 for 2 hours. Afterwards, cells were challenged with WA.1 or BA.1 at an MOI of 0.05. At 48 hours post inoculation, cells were subjected to total cellular RNA extraction and ORF1a levels were measured by RT-qPCR. Relative ORF1a transcript abundance compared to actin was determined by the 2^(-ΔΔCT) method. Symbols represent independent RT-qPCR runs. Results are represented as means plus standard error. Statistically significant differences (* *P* < 0.05) between the indicated condition and the corresponding data point of WA.1 were determined by one-way ANOVA. ns; not significant. NT; not tested (bafilomycin A1 was not tested against WA1). Cartoon made with Biorender.com.

To recreate the three-dimensional, pseudostratified architecture of nasal epithelia in vivo, primary human nasal epithelial cells were pooled from 14 donors and differentiated at the air-liquid interface (ALI). Differentiation status was confirmed by stratification of nuclei and the presence of mature cilia at the tissue surface (Figure 2A). Here, replication of Omicron BA.1 and BA.2 exceeded that of WA1 by multiple orders of magnitude, with BA.2 exhibiting the greatest replicative potential (Figure 2B). To confirm that nasal ALI support repeated rounds of productive infection, we collected culture medium supernatants of nasal ALI inoculated with WA1, BA.1, or BA.2 and quantified infectious virus levels by titrating on Vero cells. In agreement with RT-qPCR results, BA.1 and BA.2 achieved higher infectious titers in nasal ALI relative to WA1 (Figure 2C). In accordance with enhanced virus replication of BA.1 and BA.2, we detected elevated type-I interferon induction at 48 hours post-inoculation (Figure 2D). To test whether the enhanced nasal tropism of Omicron is attributed to its Spike protein, we generated recombinant SARS-CoV-2 (WA1)-mCherry that encodes Spike from WA1, BA.1, or Delta. We found that the presence of Spike from BA.1, but not Delta, resulted in a gain of nasal cell infectivity relative to WA1 (Figure 2E and F). These results indicate that enhanced nasal tissue infectivity of Omicron is governed by its Spike protein, and more specifically, by the mutations unique to Omicron Spike. Therefore, enhanced nasal cell tropism was evolved recently in the Omicron ancestor.

Since Omicron Spike encodes for an unprecedented ability to enter human nasal epithelia, we characterized the subcellular location at which Omicron Spike-mediated entry occurs and the cellular proteases that enable it. Several reports claim that Omicron enters cells via cathepsin-dependent fusion in endosomes^29,30,34^, but the Omicron entry pathway may vary in transformed cell lines.^42^ Therefore, we examined BA.1 entry determinants in primary human nasal ALI. We used E64d to disrupt cathepsin-mediated entry in endolysosomes and found that neither WA1 nor BA.1 were inhibited in nasal ALI (Figure 2G). Interestingly, BA.1 infection was enhanced 2-fold by E64d treatment, despite the same dose being previously shown to partially inhibit Omicron infection in a transformed lung cell line^29^. However, addition of camostat mesylate (an inhibitor of serine transmembrane proteases including TMPRSS2) alone or in combination with E64d strongly blocked infection of WA1 in nasal ALI (Figure 2G). This suggests that WA1 enters nasal ALI primarily via a TMPRSS2-dependent entry route at the plasma membrane. In contrast, infection of nasal ALI by BA.1 was completely insensitive to this blockade (Figure 2G), suggesting the use of a divergent entry pathway that is TMPRSS2-independent. The TMPRSS2-independence of Omicron infection has been reported by multiple groups, and as a result, it has been inferred that Omicron uses an endosomal, cathepsin-dependent route to enter human cells^29,30,34^. However, a recently described MMP-mediated entry pathway for SARS-CoV-2 that may enable virus fusion at the plasma membrane^11^ prompted us to test the effect of incyclinide, a pan-MMP inhibitor. Both WA1 and BA.1 infections were reduced by incyclinide treatment in primary ALI, but BA.1 was particularly sensitive (inhibited by 27-fold compared to 4-fold inhibition of WA1) (Figure 2G). These findings suggest that processing of BA.1 Spike by MMPs, but not TMPRSS2, enables BA.1 entry into primary nasal epithelia. To address whether this route of entry allows for fusion at the plasma membrane or at endolysosomal membranes, we blocked endosomal trafficking with the endolysosomal acidification inhibitor bafilomycin A1. Interestingly, bafilomycin A1 enhanced BA.1 infection 4-fold, suggesting that BA.1 utilizes an entry pathway that does not rely upon cathepsin activity in endolysosomes (Figure 2G). Collectively, our findings may suggest that MMP-mediated processing of BA.1 Spike enables virus entry at or near the cell surface of primary nasal epithelial cells.

**Figure 3.**
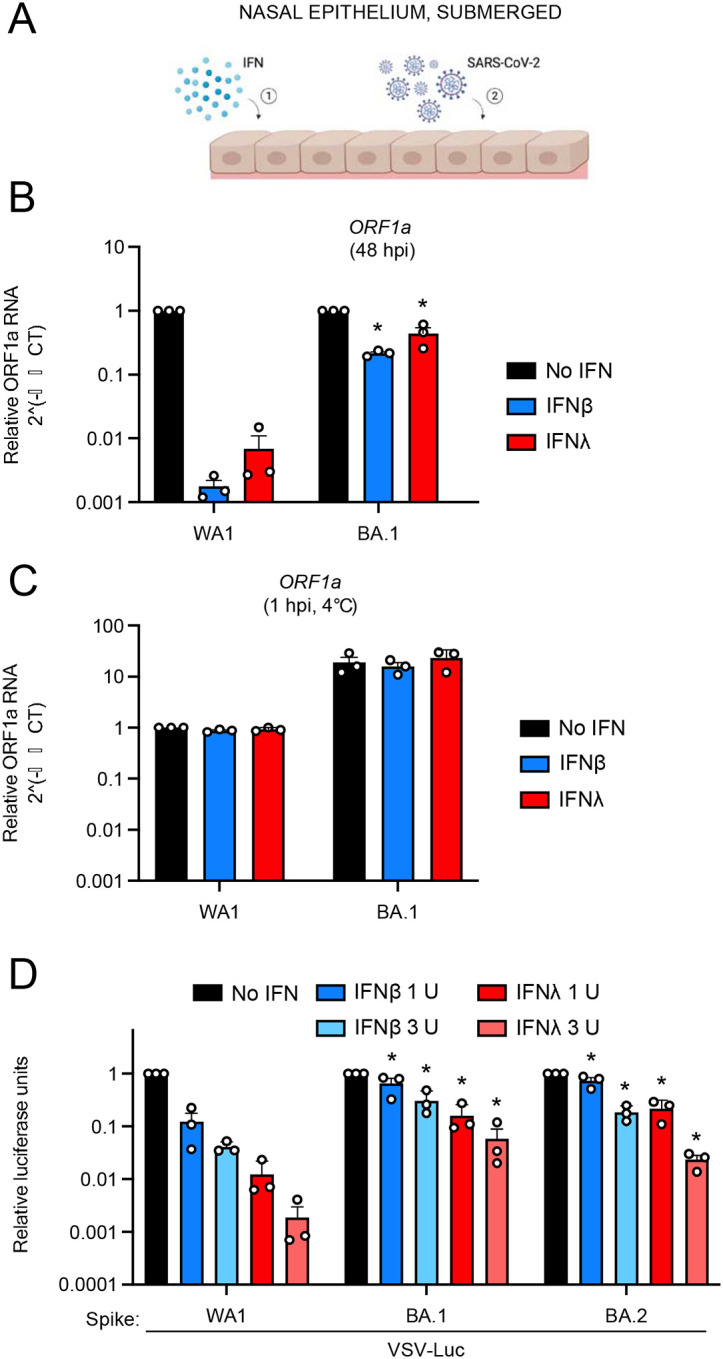
BA.1 and BA.2 entry into primary human nasal epithelia is resistant to inhibition by type-I and type-III interferons. (A) Primary human nasal epithelial cells (pooled from 3 human donors) were cultured as undifferentiated, submerged monolayers, treated with IFN-beta or IFN-lambda for 18 hours, and challenged with SARS-CoV-2. (B) Cells were pre-treated with 2 units of IFN-beta or 5 ng/mL IFN-lambda for 18 hours, inoculated with WA1 or BA.1 at an MOI of 0.025, total RNA was extracted from cells at 48 hours post inoculation, and ORF1a levels were measured by RT-qPCR. ORF1a levels were measured compared to actin using by the 2^(-ΔΔCT) method. ORF1a levels of WA1 or BA.1 in the absence of IFN were set to 1. Symbols represent results from three independent infections. (C) Cells were pre-treated with 2 units of IFN-beta or 5 ng/mL IFN-lambda for 18 hours, inoculated with WA1 or BA.1 at an MOI of 0.025 on ice, total RNA was extracted from cells at 1 hour post inoculation, and ORF1a levels were measured by RT-qPCR. ORF1a levels were measured compared to actin using by the 2^(-ΔΔCT) method. ORF1a levels of WA.1 in the absence of IFN were set to 1. Symbols represent results from three independent infections. (D) Cells were pre-treated with the indicated amounts of IFN-beta or IFN-lambda for 18 hours and challenged with VSV-based pseudovirus decorated with Spike from WA1, BA.1, or BA.2. At 24 hours post inoculation, luciferase activity was measured from lysed cells. Luciferase activity of WA1, BA.1, and BA.2 pseudoviruses in the absence of IFN were set to 1. Symbols represent results from three independent infections. Results are represented as means plus standard error. Statistically significant differences (* *P* < 0.05) between the indicated condition and the corresponding data point of WA.1 were determined by one-way ANOVA. Cartoon made with Biorender.com.

So far, we have shown that Omicron Spike gains access to primary nasal epithelia by improved adhesion to the cell surface and entry/fusion triggered by MMP activity. During the preparation of this manuscript, it was demonstrated that SARS-CoV-2 variants of concern displayed an increasing resistance to inhibition by interferon (IFN) treatment, with Omicron exhibiting the greatest degree of resistance^43^. However, the role of Spike in conferring IFN resistance was not assessed, and it was unknown whether an Omicron-specific cellular entry pathway enabled IFN resistance in nasal cells. Therefore, we tested whether the unique entry requirements of Omicron Spike allow for evasion of the IFN-induced antiviral state. We pre-treated primary undifferentiated nasal monolayers pooled from three human donors with type-I IFN (IFN-beta) or type-III IFN (IFN-lambda) and challenged cells with WA1 or BA.1 (Figure 3A). While IFN-beta exposure strongly blocked WA1 infection by several thousand-fold, the same amount of IFN inhibited BA.1 to a much lesser extent (less than 10-fold) (Figure 3B). Similarly, IFN-lambda potently blocked WA1 infection but was much less effective against BA.1 (Figure 3B). We determined that cellular attachment of WA1 and BA.1 was not affected by treatment by IFN-beta or IFN-lambda, ruling out that Omicron escapes interferon-induced cellular factors that restrict the attachment step (Figure 3C). To formally assess the role of Spike in the resistance of Omicron to type-I and type-III interferons, we generated pseudovirus consisting of vesicular stomatitis virus (VSV) decorated with SARS-CoV-2 Spike variants. VSV-WA1 Spike was exquisitely sensitive to inhibition by IFN-beta or IFN-lambda in a dose-dependent manner, with inhibition up to two orders of magnitude observed. In contrast, VSV-BA.1 Spike and VSV-BA.2 Spike were much less sensitive to inhibition by IFN-beta or IFN-lambda (Figure 3D). Therefore, Spike is a major determinant of the IFN resistance displayed by Omicron, and this feature can be transferred to other viruses by pseudotyping.

**Figure 4.**
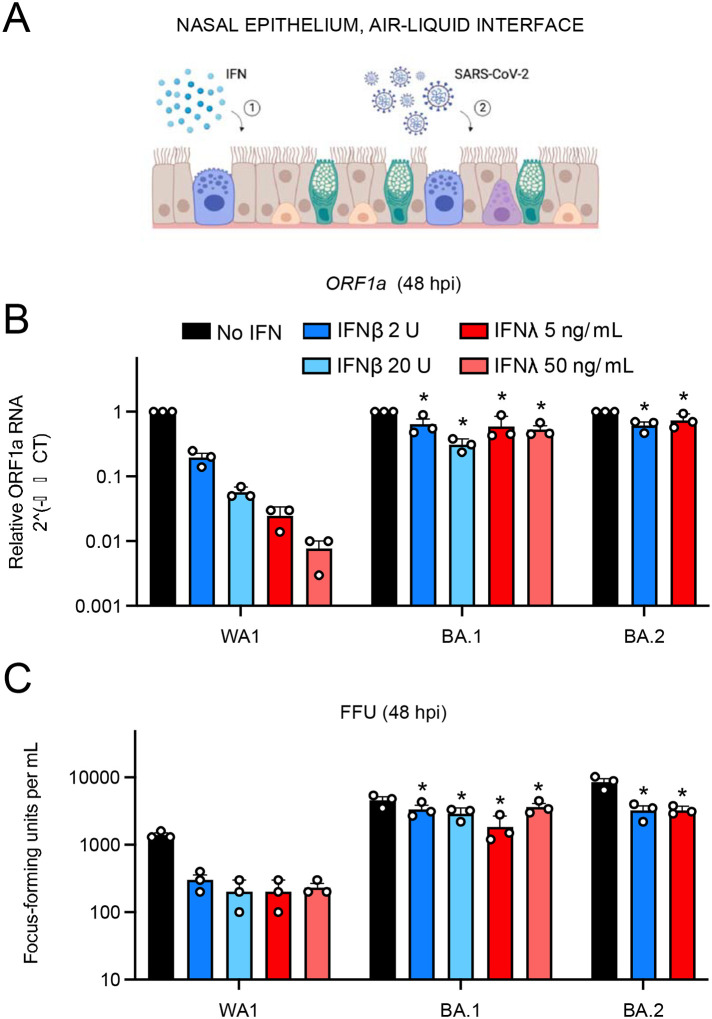
Entry of BA.1 and BA.2 into primary nasal epithelial cells cultured at the air-liquid interface is resistant to inhibition by type-I and type-III interferons. (A) Primary human nasal epithelial cells (pooled from 14 human donors) were cultured at the air-liquid interface, pre-treated for 18 hours with the indicated amounts of IFN-beta or IFN-lambda, and challenged with 10000 plaque forming units of WA1, BA.1, or BA.2. (B) At 48 hours post inoculation, total RNA was extracted from cells and subjected to RT-qPCR. ORF1a levels were measured compared to actin using by the 2^(-ΔΔCT) method. Symbols represent independent RT-qPCR runs. (C) Infectious virus titers from cell culture medium were measured by challenging Vero cells followed by fixation and immunostaining with anti-N antibody. Infectious units were quantified by measuring focus forming units by high-content imaging. Symbols represent independent infections of Vero cells. Results are represented as means plus standard error. Statistically significant differences (* *P* < 0.05) between the indicated condition and the corresponding data point of WA.1 were determined by one-way ANOVA. Cartoon made with Biorender.com.

Next, we addressed whether Omicron subvariants display resistance to the antiviral state in primary nasal ALI (Figure 4A). As was observed in nasal monolayers, pre-treatment with IFN-beta or IFN-lambda inhibited WA1 in a dose-dependent manner (up to two orders of magnitude) as measured by viral RT-qPCR, while BA.1 or BA.2 infections were considerably less impacted (Figure 4B). We also quantified infectious virus yield under these conditions and confirmed that BA.1 and BA.2 achieved higher titers and were inhibited by IFN to a lesser extent than WA1 (Figure 4C).

Overall, by combining experiments with authentic SARS-CoV-2, recombinant virus, and Spike-decorated pseudovirus, we show that Spike is the major determinant of the enhanced infectivity and interferon resistance exhibited by Omicron in nasal epithelia, two phenotypes which are associated with a unique dependence on MMPs for cell entry at or near the cell surface. Moreover, our results reveal the existence of type-I- and type-III IFN-inducible host factors in primary nasal epithelia that restrict Spike-mediated entry at a post-attachment step.

## Discussion

Using entirely primary nasal epithelia from human adult donors, we demonstrate that Omicron (including BA.1 and BA.2) exhibits drastically increased infectivity in nasal tissue compared to ancestral SARS-CoV-2 and the variant of concern that preceded it, Delta. This mapped to Omicron Spike and, at least in part, to an increased ability for Omicron virions to adhere to the surface of nasal epithelia, which occurred independently of the presence of cilia. Furthermore, we show that Omicron utilizes an entry route into cells that depends on cellular MMPs but that is independent of the transmembrane serine protease TMPRSS2. Since we also demonstrate that Omicron Spike-mediated infection enables evasion of the antiviral state induced by type-I and type-III IFN in this tissue, these results suggest that the MMP-dependent entry pathway utilized by Omicron may promote escape from IFN-induced host factors targeting entry.

During the preparation of this manuscript, it was reported that BA.1 exhibits a growth advantage in primary human nasal epithelia compared to the Delta variant^29^. Here, using recombinant SARS-CoV-2, we extend this finding by demonstrating that Omicron Spike is responsible for increased nasal cell infectivity. However, in another study, while BA.1 exhibited a gain in nasal cell infectivity compared to Alpha, no significant differences in nasal cell infectivity were observed between Delta and BA.1^30^. This discrepancy is likely the result of utilization of distinct cell culture models to compare relative infectivities. Our use of recombinant, fluorogenic SARS-CoV-2 in nasal ALI derived from pools of 14 human adult donors shows that Spike from BA.1 promotes a gain in nasal cell infectivity compared to Delta Spike. Therefore, the emergence and evolution of Omicron involved unique mutations in Spike that contributed to enhanced nasal cell binding and entry, and this may explain how Omicron replaced previously circulating variants of concern and continues to persist in human populations.

It was previously reported that human nasal airways express relatively low levels of TMPRSS2, and combined with the demonstration that Omicron exhibits TMPRSS2-independence in transformed cell lines, others have inferred that Omicron is more reliant on endosomal cathepsins for entry^30^. However, our findings with primary nasal epithelia reveal that TMPRSS2-independence does not necessarily imply the use of an endosomal entry route by Omicron. MMPs such as MMP14 and MMP16 are also highly expressed in upper airways and have been shown to activate Omicron Spike-mediated fusion at the surface of several transformed cell lines^11^. Here, we demonstrate that the primary route of entry of Omicron in primary nasal epithelia is mediated by MMPs. The pan-MMP inhibitor incyclinide strongly blocked BA.1 infection in nasal ALI, but the endosomal cathepsin inhibitor E64d and the endolysosomal acidification inhibitor bafilomycin A1 did not. Instead, these endosomal inhibitors boosted infection of BA.1 in nasal epithelia, suggesting that the endosomal entry route is deleterious for BA.1 in this tissue. Bafilomycin A1 inhibits vacuolar-ATPase to prevent endolysosomal acidification, and furthermore, endosomal maturation and transport between early and late endosomes is repressed^44^. Therefore, our results suggest that processing of Spike by MMPs enables fusion at the plasma membrane and/or in early or recycling endosomes, while late endosomes or lysosomes and the cathepsins found therein are unlikely to be involved.

One possibility explaining the enhanced capacity for Omicron Spike to adhere to and enter nasal cells (either in the presence or absence of ciliated cells) is its increase in net overall positive charge compared to previous variants of concern^45^. It has also been proposed that Omicron Spike may adhere more strongly to attachment factors present at the epithelial cell surface, including heparan sulfate^46,47^ and neurolipin 1^48^. A significant aim of future research will be to establish whether mutations in Omicron Spike promote increased residence time at the cell surface and whether this influences its dependence on certain cellular proteases for cleavage and the triggering of fusion. It is possible that an improved capacity for Omicron Spike to bind heparan sulfate and neurolipin 1 may negatively interfere with processing by TMPRSS2 and steer processing towards MMPs such as MMP14 and MMP16.

Another factor that likely contributed to the spread and dominance of Omicron lineages is their resistance to the antiviral properties of IFN. Early/ancestral SARS-CoV-2 was previously reported to antagonize IFN signaling in cells, and nearly every viral protein encoded by SARS-CoV-2 has been shown to contribute to evasion of IFN through various mechanisms^49–51^. While the relative insensitivity of Omicron to exogenous administration of multiple IFN types has been reported previously^43,52^, the viral proteins responsible for rendering IFN ineffective against Omicron were unknown. However, other studies showed that Omicron exhibits a decreased capacity to interfere with IFN production and signaling, relative to early/ancestral SARS-CoV-2 or Delta,^42,53–55^ and our measurements of type-I IFN production by Omicron-infected nasal ALI affirm this finding. However, in one case, Omicron was purported to induce a relatively inferior IFN response compared to Delta^52^, and this discrepancy underlines the importance of assessing this activity in primary human upper airway epithelia. Overall, we postulate that Omicron exhibits resistance to IFN not because of its ability to interfere with the production or function of IFN from infected cells, but rather, because Omicron Spike enables passage into nasal cells in a manner which is insensitive to IFN-stimulated proteins. In addition, it is possible that Omicron displays enhanced infectivity in nasal cells even in the absence of IFN because Omicron Spike confers resistance to antiviral factors that are constitutively expressed in nasal epithelia and further upregulated by type-I or type-III IFN. Furthermore, our findings provide a new explanation for why Omicron exhibits decreased IFN antagonism—if the Spike-mediated entry process of Omicron is resistant to type-I and type-III IFN, the selective advantage to antagonize IFN pathways may be lost. Furthermore, the improved capacity for Omicron lineages to infect nasal cells despite the induction of an antiviral state therein provides a plausible scenario for how Omicron displaced previous variants of concern. It was demonstrated that IFN produced from Omicron-infected cells can inhibit infection by Influenza A virus^53^, so it is plausible that the IFN response elicited by Omicron infection may render nasal epithelia resistant to infection by previous SARS-CoV-2 variants of concern. Indeed, it has been shown that Omicron displays a greater degree of IFN-resistance compared to Delta^43^.

Our description of the IFN resistance conferred by Omicron Spike has implications for the ongoing development of type-I and type-III IFN as antiviral therapeutics for the prevention or treatment of SARS-CoV-2 infection and COVID-19. A case has been made for the deployment of type-III IFN in the context of SARS-CoV-2 infection, since the receptor for type-III IFN is expressed in the respiratory tract but is not widely distributed elsewhere (resulting in fewer unintended side effects such as inflammation)^49,56^. As an important proof-of-concept, nasal administration of IFN-lambda was protective against SARS-CoV-2 variants, including Omicron, in mice^57^. However, clinical trials thus far in humans have reported conflicting findings. One such study reported that participants who were administered a single injection of IFN-lambda following symptom onset showed no significant reductions in viral shedding or symptom severity compared to placebo,^58^ while another reported that a single injection upon symptom onset resulted in reduced viral loads^59^ and reduced COVID-19-related hospitalizations.^60^ However, in neither case was it determined how IFN-lambda fared in individuals infected with Omicron variants. Since Omicron displays a greater degree of IFN-resistance compared to earlier variants, additional clinical studies are needed to test the suitability of IFN-lambda in the fight against contemporaneous Omicron and additional variants that arise in the future.

Overall, our findings suggest that mutations in Omicron Spike, while enabling evasion of vaccine-elicited neutralizing antibodies, also allow for efficient seeding of infection in the nasal epithelium and resistance to the interferon-induced antiviral state therein. These results provide mechanistic insight into the basis for the efficient transmission and persistence of Omicron in human populations.

## Materials and Methods

### Tissue culture

Frozen primary human nasal airway epithelial cells (hNAEC) were purchased from Epithelix and cultured as submerged monolayers with hAEC Culture Medium (Epithelix). hNAEC from three human donors were thawed and pooled, and cells were passed every 5 days. Fresh primary human nasal airway epithelial cells cultured at the liquid-air interface (nasal ALI) were obtained from Epithelix (MucilAir, Pool of Donors) and cultured with MucilAir culture medium (Epithelix) in trans-well plates provided by the company; culture medium was replaced every 2 days. Nasal ALI cultures were maintained for 2–5 weeks in our laboratories prior to inoculation with SARS-CoV-2. hNAEC and Nasal ALI were fixed with 4% paraformaldehyde, permeabilized with 0.1% Triton-X, and stained with anti-Acetyl-a-Tubulin (Lys40) (D20G3) (Cell Signaling; 5335) and DAPI to assess pseudostratification and the presence of ciliated cells. Images were obtained using the Zeiss LSM 880 workstation and AiryScan capability and were processed into 3D reconstructions by volume rendering using Imaris software. Primary human small airway (lung) epithelial cells (hSAEC) from a single human donor were purchased from ATCC (PCS-301–010) and were cultured with Airway Epithelial Cell Basal Medium (ATCC, PCS-300–030) and the Bronchial Epithelial Growth Kit (ATCC, PCS- 300–400). Vero E6 were purchased from ATCC (CRL-1586) and Vero E6-TMPRSS2 cells were generated by introducing the TMPRSS2 open reading frame using the Sleeping Beauty transposon system.

### Production of SARS-CoV-2 variants and infections

SARS-CoV-2 isolate USA/WA1/2020 (NR-52281), isolate hCoV-19/USA/HI-CDC-4359259–001/2021 (Lineage B.1.1.529; Omicron BA.1 variant; NR-56475), and isolate hCoV-19/USA/NY-MSHSPSP-PV56475/2022 (Lineage BA.2.12.1; Omicron BA.2 variant; NR-56782) were obtained from BEI Resources. Recombinant WA1-mCherry virus was rescued from a SARS-CoV-2 cDNA construct^61^, and mCherry was inserted at the N-terminus of the *N* gene. A P2A linker was placed between mCherry and N. To generate WA1-mCherry (BA.1 Spike) and WA1-mCherry (Delta Spike), the sequence encoding WA1 Spike was replaced with Spike from BA.1 or Delta, respectively. All viruses were grown in Vero E6-TMPRSS2 cells and harvested cell culture supernatants were titrated in Vero E6 to calculate infectious titers.

Infections of hNAEC or hSAEC monolayers were performed at a multiplicity of infection (MOI) of 0.05 as follows: cells were inoculated with virus suspension for 2 hours at 37°C; inoculum was removed; cells were washed with PBS and returned to 37°C for the time indicated. For detection of virus attachment, hNAEC were inoculated with virus suspension for 1 hour on ice; inoculum was removed; cells were washed three times with cold PBS.

For detection of infection or virus attachment by RT-qPCR, cells were lysed with Trizol (Sigma). Viral replication was measured using RT-qPCR amplification of viral ORF1a, as previously described^62^. Cells lysed with Trizol were mixed with chloroform (Sigma) at a 5:1 (Trizol:chloroform) ratio. Mixed samples were mixed thoroughly and incubated at room temperature for 10 minutes, followed by centrifugation at 12000 × G for 5 minutes to allow separation of the aqueous and organic phases. Equal volumes of 70% ethanol were added to the aqueous phases, mixed thoroughly, and incubated at room temperature for 5 minutes. RNA purification was performed using the PureLink RNA Mini Kit (Invitrogen) according to manufacturer’s instructions. Purified RNA product was immediately used with the One-step PrimeScript RT-PCR Kit (Takara). Primers and probes were obtained from IDT. The primers and probes used to amplify and quantify ORF1a are as follows (5’−3’): ORF1a-F AGAAGATTGGTTAGATGATGATAGT; ORF1a-R TTCCATCTCTAATTGAGGTTGAACC; ORF1a-P FAM/TCCTCACTGCCGTCTTGTTGACCA/BHQ13. The primers and probes used to amplify and quantify beta actin (ACTB) are as follows (5’−3’): ACTB-F ACAGAGCCTCGCCTTTG; ACTB-R CCTTGCACATGCCGGAG; ACTB-P 56-FAM/TCATCCATG/ZEN/GTGAGCTGGCGG/31ABkFQ. Reaction mixtures of 20 μL (including 2.2 μL total RNA, 0.2 μM forward and reverse primers, and 0.1 μM probe) were subjected to reverse transcription (5 min at 45°C, followed by 10 s at 95°C) and 40 cycles of PCR (5 s at 95°C followed by 20 s at 60°C) in a CFX Opus 96 Real-Time PCR System (BioRad). Results were analyzed by the Comparative Ct Method (ΔΔCt Method)^63^. RNA levels for viral ORF1a were normalized to cellular ACTB.

For Infections of primary nasal epithelial cells cultured at the air-liquid interface, the apical (air-exposed) surface was gently rinsed with 100 μL cell culture medium to partially remove mucous layers. Virus suspension in a volume of 50 μL was added to the apical surface and cells were incubated for 2 hours at 37°C. Inoculum was then removed, 100 μL PBS was used to gently wash the apical surface. Cells were then returned to 37°C. At 48 hours post inoculation, cells were lysed with Trizol and subjected to RNA extraction and RT-qPCR as indicated above. To quantify the infectious virus particles produced into the cell culture supernatant, medium was removed, and cells were gently washed with 100 μL PBS to recover virus. A focus-forming units assay was performed by inoculating Vero cells with the recovered volume. At 7 hours post inoculation, Vero cells were fixed with 4% paraformaldehyde and stained with anti-nucleocapsid antibody recognizing SARS-CoV-2 nucleocapsid (Invitrogen, MA1–7403) and a secondary antibody coupled to AlexaFluor-488 (Invitrogen). The number of fluorescent foci was measured using a Cytation 5 Cell Imaging Multimode Reader (BioTeK).

For detection of recombinant WA-mCherry infection, the Cytation 5 Cell Imaging Multimode Reader (BioTeK) was used to measure mCherry fluorescence at 24 hours post inoculation. Quantification of mCherry fluorescence was performed using Fiji.

### Production of VSV-Spike pseudoviruses and infections

Spike sequences from WA.1, BA.1, or BA.2 were codon-optimized for expression in human cells, synthesized with a 6xHis tag on the amino-terminus, and cloned into pcDNA3.1 (+) by GenScript. HEK293T cells were seeded in a 10 cm dish and transfected with 12 μg pcDNA3.1 Spike plasmid using Lipofectamine 2000 (Thermo Fisher). Forty-eight hours after transfection, culture medium was removed from cells, and 1 mL VSV-luc/GFP plus VSV-G (seed particles) was added. Twenty-four hours after infection, virus supernatants were collected, clarified by centrifugation at 500*g* for 5 minutes followed by filtration with a 45 μm filter, and stored at - 80°C. A total of 50 μL virus supernatants was added to submerged hNAEC from three pooled human donors, and 24 hours post inoculation, cells were lysed with Passive Lysis Buffer (Promega). Luciferase activity was measured on a Perkin Elmer MicroBeta 2450 microplate luminometer using the Luciferase Assay System (Promega). 50 μL volumes of VSV-WA1, VSV-BA.1, and VSV-BA.2 were found to infect Vero E6 cells to similar extents, suggestive of similar infectious titers of these pseudoviruses.

### Interferons and Inhibitors

Recombinant human IFN-β (beta) (300–02BC) and human IFN-λ (lambda) (300–02L) were obtained from PeproTech and were used to test IFN sensitivity of full-length, authentic SARS-CoV-2 variants (IFN was added 24 hours prior to inoculation and removed prior to virus addition). Recombinant human IFN-β (beta) 1a (11415–1) and human IFN-λ (lambda) (11725–1) were obtained from PBL Assay Science and were used to test IFN sensitivity of VSV-based pseudoviruses (IFN was added 24 hours prior to inoculation and removed prior to virus addition). The protease inhibitor E64d (E8640) was obtained from Sigma and reconstituted in DMSO. The serine transmembrane protease inhibitor camostat mesylate (SML0057) was obtained from Sigma and reconstituted in DMSO. The MMP inhibitor incyclinide (HY-13648) was obtained from MedChemExpress. The endolysosomal acidification inhibitor bafilomycin A1 (SML1661) was obtained from Sigma as a ready-made solution in DMSO.

### Biosafety Approval

This study was conducted in compliance with all relevant local, state, and federal regulations. Approval for the generation and use of recombinant SARS-CoV-2 WA1-mCherry and its variants using Biosafety Level 3 practices was provided by the NIAID Institutional Biosafety Committee following evaluation by the Dual Use Research of Concern Institutional Review Entity (case number RD-22-X1–11; PI: Jonathan W Yewdell).
