# Supplementary material for: Omicron Spike confers enhanced infectivity and interferon resistance to SARS-CoV-2 in human nasal tissue
Source: bioRxiv. 2023 Oct 12:2023.05.06.539698. Originally published 2023 May 8. Preprint. [Version 2] doi: 10.1101/2023.05.06.539698 (PMC10327209; doi:10.1101/2023.05.06.539698)
Supplement: Supplement 1 [file NIHPP2023.05.06.539698v2-supplement-1.pdf]

bioRxiv preprint doi: https://doi.org/10.1101/2023.05.06.539698; this version posted October 12, 2023. The copyright holder for this preprint (which was not certified by peer review) is the author/funder. This article is a US Government work. It is not subject to copyright under 17 USC 105 and is also made available for use under a CC0 license.

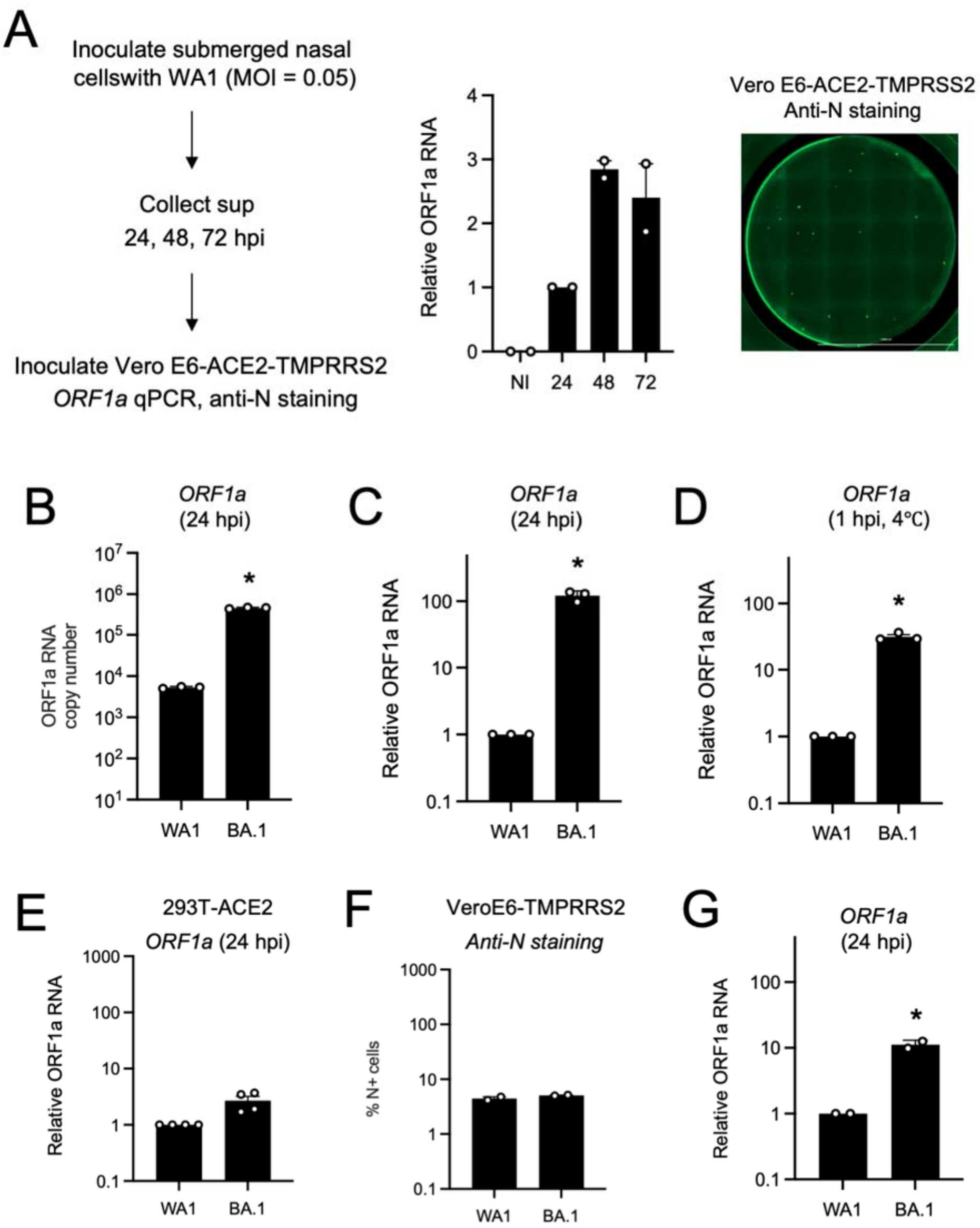

Supplemental Figure 1.

(A) Primary human nasal epithelial cells (pooled from 3 human donors) were cultured as undifferentiated, submerged monolayers and challenged with WA1 at an MOI of 0.05. Cell culture supernatants were collected at 24, 48, and 72 hours post-inoculation and added to Vero E6-ACE2-TMPRSS2 cells. 24 hours later, Vero E6-ACE2-TMPRSS2 cells were subjected to total RNA extraction and viral ORF1a RT-qPCR was performed. In addition, productive infection of Vero E6-ACE2-TMPRSS2 by the 24 hours post-inoculation supernatant was confirmed by anti-N immunofluorescence microscopy. (B) Primary human nasal epithelial cells (cells from three human donors, pooled) were inoculated with WA1 or BA.1 at an MOI of 0.05.

bioRxiv preprint doi: https://doi.org/10.1101/2023.05.06.539698; this version posted October 12, 2023. The copyright holder for this preprint (which was not certified by peer review) is the author/funder. This article is a US Government work. It is not subject to copyright under 17 USC 105 and is also made available for use under a CC0 license.

Total cellular RNA was extracted and viral ORF1a was quantified by RT-qPCR at 24 hours post-inoculation. Absolute ORF1a RNA copy numbers were calculated by comparison to an ORF1a standard curve. (C) Relative viral ORF1a RNA abundance compared to actin was determined by the 2^(-ΔΔCT) method. ORF1a abundance of WA1 was set to 1. (D) Primary human nasal epithelial cells (cells from three human donors, pooled) were inoculated with WA1 or BA.1 at an MOI of 0.05 on ice. At 1 hour post-inoculation, total cellular RNA was extracted and viral ORF1a was quantified by RT-qPCR to measure virus adherence to cells. Relative viral ORF1a RNA abundance compared to actin was determined by the 2^(-ΔΔCT) method. ORF1a abundance of WA1 was set to 1. (E) HEK293T-ACE2 cells were inoculated with WA1 or BA.1 at an MOI of 0.05. 24 hours post-inoculation, total cellular RNA was extracted and viral ORF1a was quantified by RT-qPCR. Relative viral ORF1a RNA abundance compared to actin was determined by the 2^(-ΔΔCT) method. ORF1a abundance of WA1 was set to 1. (F) Vero E6-TMPRSS2 cells were inoculated with WA1 or BA.1 at an MOI of 0.05. 24 hours post-inoculation, cells were fixed, stained with anti-N antibody, and infection was scored by flow cytometry. (G) Primary human nasal epithelial cells (cells from three human donors, pooled) were inoculated with WA1 or BA.1 (5x10^7 copies of absolute ORF1a RNA used as input). 24 hours post-inoculation, total cellular RNA was extracted and viral ORF1a was quantified by RT-qPCR. Relative viral ORF1a RNA abundance compared to actin was determined by the 2^(-ΔΔCT) method. ORF1a abundance of WA1 was set to 1. All results are represented as means plus standard error from three independent infections (symbols represent biological replicates). Statistically significant differences (* $P < 0.05$) between the indicated condition of BA.1 and the corresponding condition of WA.1 were determined by one-way ANOVA.

bioRxiv preprint doi: https://doi.org/10.1101/2023.05.06.539698; this version posted October 12, 2023. The copyright holder for this preprint (which was not certified by peer review) is the author/funder. This article is a US Government work. It is not subject to copyright under 17 USC 105 and is also made available for use under a CC0 license.

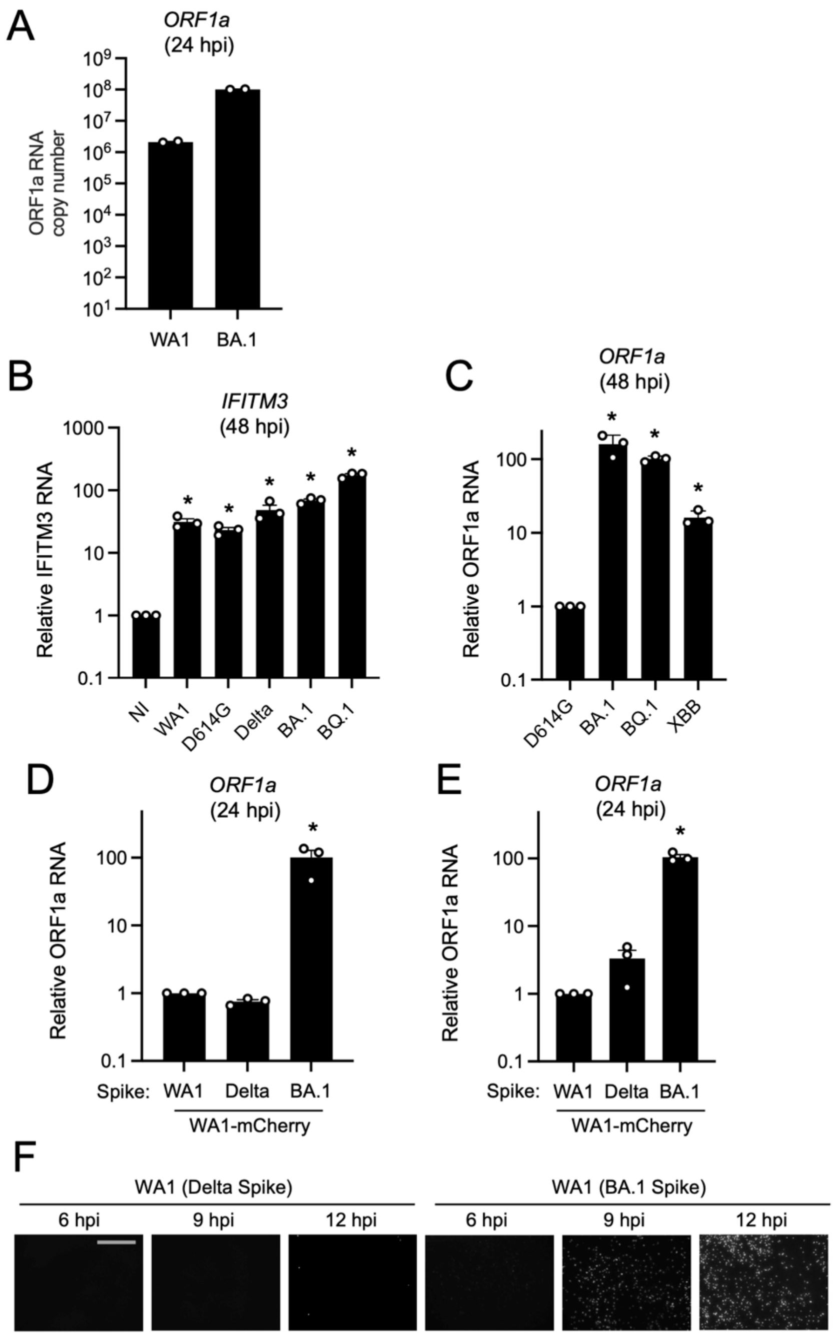

Supplemental Figure 2.

(A) Primary human nasal epithelial cells (pooled from 14 human donors) were cultured at the air-liquid interface and inoculated with 10000 plaque forming units of WA1 or BA.1. Total cellular RNA was extracted and viral ORF1a was quantified by RT-qPCR at 24 hours post-inoculation. Absolute ORF1a RNA copy numbers were calculated by comparison to an ORF1a standard curve. (B) Primary human nasal epithelial cells (pooled from 14 human donors) were cultured at the air-liquid interface and inoculated with 10000 plaque forming units of WA1, D614G, Delta, BA.1, or BQ.1. RT-qPCR of cellular *IFITM3* was performed at 48 hours post inoculation. Relative *IFITM3* transcript abundance was compared to actin using the 2^(-ΔΔCT)

bioRxiv preprint doi: https://doi.org/10.1101/2023.05.06.539698; this version posted October 12, 2023. The copyright holder for this preprint (which was not certified by peer review) is the author/funder. This article is a US Government work. It is not subject to copyright under 17 USC 105 and is also made available for use under a CC0 license.

method. *IFITM3* abundance in non-inoculated (NI) cells was set to 1. Statistically significant differences (* $P < 0.05$) between the indicated condition and the NI condition were determined by one-way ANOVA. (C) Primary human nasal epithelial cells (pooled from 14 human donors) were cultured at the air-liquid interface and inoculated with 10000 plaque forming units of D614G, BA.1, BQ.1, or XBB. At 48 hours post-inoculation, total cellular RNA was extracted and viral ORF1a was quantified by RT-qPCR. Relative ORF1a abundance was determined by comparing to actin using the 2^(-ΔΔCT) method. Statistically significant differences (* $P < 0.05$) between the indicated condition and D614G were determined by one-way ANOVA. (D) 10000 plaque forming units of recombinant WA.1 encoding mCherry and Spike protein from WA1, Delta, or BA.1 (WA1-mCherry (WA1 Spike), WA1-mCherry (Delta Spike), and WA1-mCherry (BA.1 Spike)) were used to inoculate primary human nasal epithelial cells (pooled from 14 human donors) cultured at the air-liquid interface. At 48 hours post-inoculation, total cellular RNA was extracted and viral ORF1a was quantified by RT-qPCR. Relative ORF1a abundance was determined by comparing to actin using the 2^(-ΔΔCT) method. Statistically significant differences (* $P < 0.05$) between the indicated condition and WA1-mCherry (WA1 Spike) were determined by one-way ANOVA. (E) As in (D), except that 5x10^7 copies of absolute ORF1a RNA were used as input. At 48 hours post-inoculation, infection was measured by viral ORF1a RT-qPCR. Relative ORF1a abundance was determined by comparing to actin using the 2^(-ΔΔCT) method. Statistically significant differences (* $P < 0.05$) between the indicated condition WA.1 were determined by one-way ANOVA. All results are represented as means plus standard error from three independent infections (symbols represent biological replicates). (F) 10000 plaque forming units of recombinant WA.1 encoding mCherry and Spike protein from Delta or BA.1 (WA1-mCherry (Delta Spike), and WA1-mCherry (BA.1 Spike)) were used to inoculate primary human nasal epithelial cells (pooled from 14 human donors) cultured at the air-liquid interface. At 6, 9, and 12 hours post-inoculation, infection was measured by mCherry fluorescence. Scale bar = 300 microns.

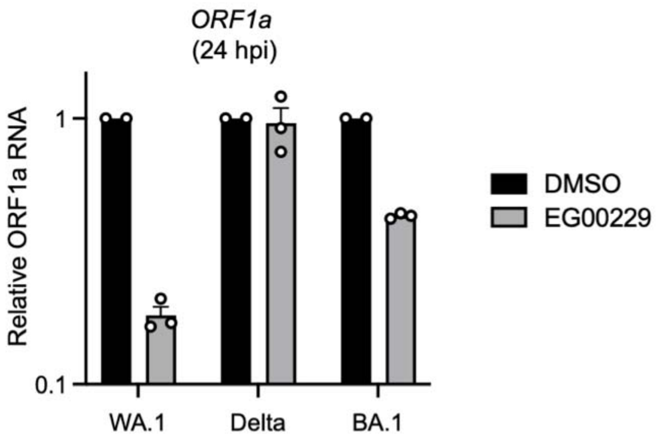

Supplemental Figure 3.

Primary human nasal epithelial cells (pooled from 14 human donors) were cultured at the air-liquid interface, pre-treated with 100 μM EG00229 or DMSO for two hours, and inoculated with

bioRxiv preprint doi: https://doi.org/10.1101/2023.05.06.539698; this version posted October 12, 2023. The copyright holder for this preprint (which was not certified by peer review) is the author/funder. This article is a US Government work. It is not subject to copyright under 17 USC 105 and is also made available for use under a CC0 license.

10000 plaque forming units of WA1, Delta, or BA.1. mCherry fluorescence was measured at 24 hours post-inoculation by high-content imaging. The fluorescence intensity of the DMSO-treated condition for each virus was set to 1. Scale bar = 300 µm. Results are represented as means plus standard error from one infection (symbols represent three RT-qPCR runs).

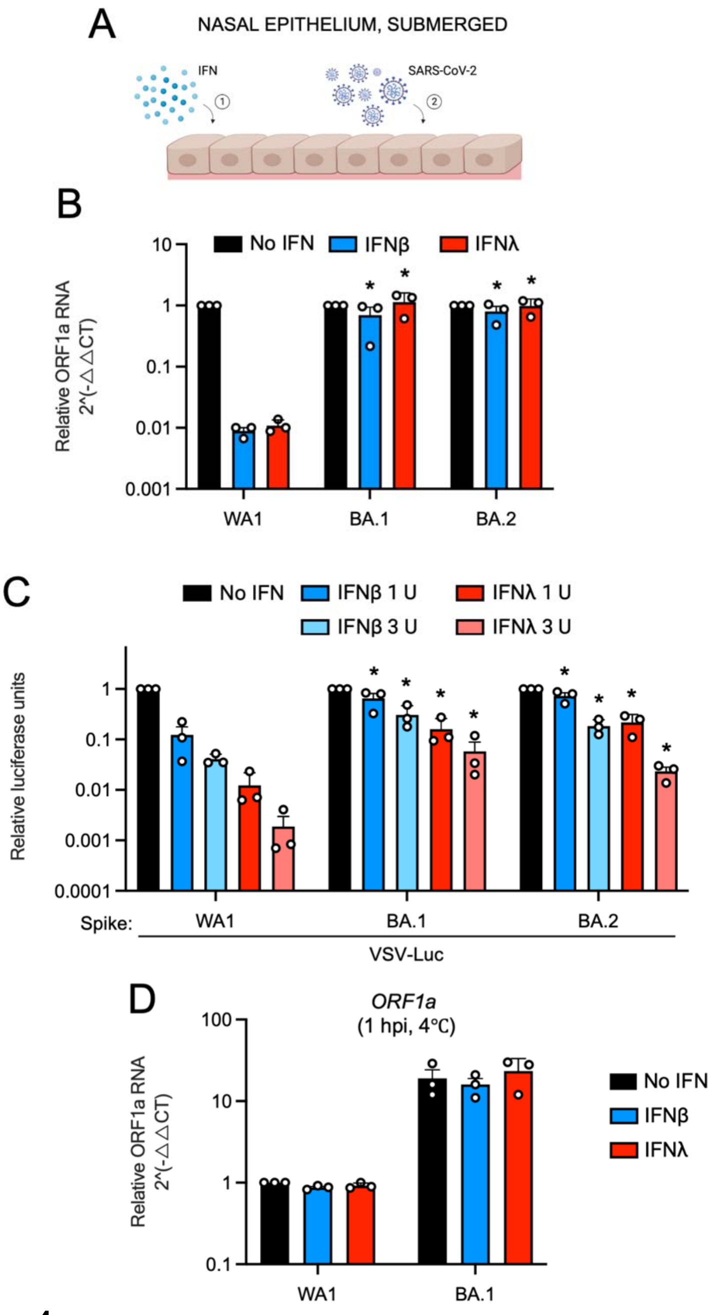

Supplemental Figure 4.

(A) Primary human nasal epithelial cells (pooled from 3 human donors) were cultured as undifferentiated, submerged monolayers, treated with IFN-beta or IFN-lambda for 18 hours, and challenged with SARS-CoV-2. Cartoon made with Biorender.com. (B) Cells were pre-treated

bioRxiv preprint doi: https://doi.org/10.1101/2023.05.06.539698; this version posted October 12, 2023. The copyright holder for this preprint (which was not certified by peer review) is the author/funder. This article is a US Government work. It is not subject to copyright under 17 USC 105 and is also made available for use under a CC0 license.

with 2 units of IFN-beta or 2 units of FN-lambda for 18 hours, and inoculated with WA1, BA.1, or BA.2 at an MOI of 0.05. Total RNA was extracted from cells at 24 hours post inoculation, and ORF1a levels were measured by RT-qPCR. Relative ORF1a abundance was determined by comparing to actin using the 2^(-ΔΔCT) method. For each virus, ORF1a levels in the absence of IFN were set to 1. (C) Primary human nasal epithelial cells (pooled from 3 human donors) were pre-treated with the indicated amounts of IFN-beta or IFN-lambda for 18 hours and challenged with VSV-based pseudovirus decorated with Spike from WA1, BA.1, or BA.2. At 24 hours post inoculation, luciferase activity was measured from lysed cells. Luciferase activity of WA1, BA.1, and BA.2 pseudoviruses in the absence of IFN were set to 1. (D) Primary human nasal epithelial cells (pooled from 3 human donors) were pre-treated with 2 units of IFN-beta or 5 ng/mL IFN-lambda for 18 hours, and inoculated with WA1 or BA.1 at an MOI of 0.05 on ice. Total RNA was extracted from cells at 1 hour post inoculation, and ORF1a levels were measured by RT-qPCR. Relative ORF1a abundance was determined by comparing to actin using the 2^(-ΔΔCT) method. ORF1a levels of WA.1 in the absence of IFN were set to 1. All results are represented as means plus standard error from three independent infections (symbols represent biological replicates). Statistically significant differences (* $P < 0.05$) between the indicated condition and the corresponding No IFN condition were determined by one-way ANOVA.

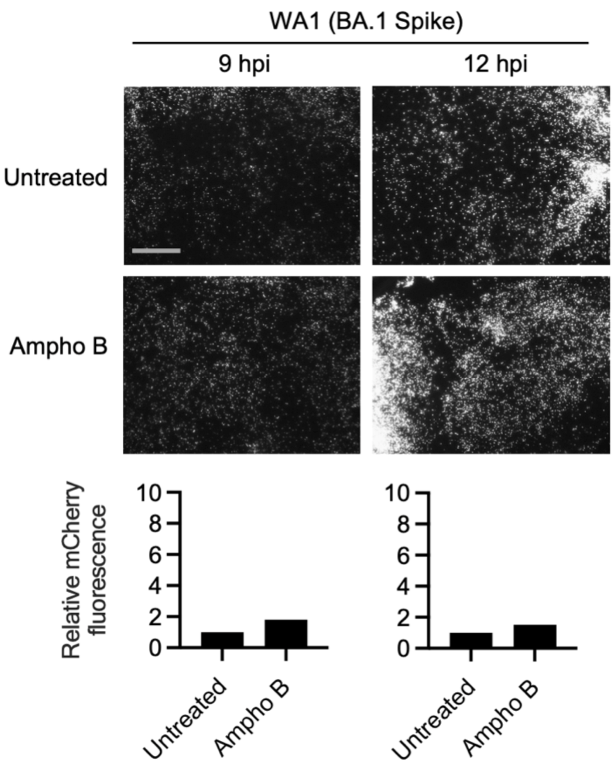

Supplemental Figure 5.

Primary human nasal epithelial cells (pooled from 14 human donors) were cultured at the air-liquid interface, pre-treated with 1 µM Amphotericin B for two hours or untreated, and inoculated with 10000 plaque forming units of WA1-mCherry (BA.1 Spike). mCherry fluorescence was measured at 9 and 12 hours post-inoculation by high-content imaging. The

bioRxiv preprint doi: https://doi.org/10.1101/2023.05.06.539698; this version posted October 12, 2023. The copyright holder for this preprint (which was not certified by peer review) is the author/funder. This article is a US Government work. It is not subject to copyright under 17 USC 105 and is also made available for use under a CC0 license.

fluorescence intensity of the untreated condition was set to 1. Scale bar = 300 μm. Ampho B; amphotericin B.
